# Ovarian Juvenile Granulosa Cell Tumor Case Report

**DOI:** 10.21980/J8035H

**Published:** 2022-01-15

**Authors:** Jasmine Lemmons, Kim Little-Wienert, Alia Hamad

**Affiliations:** * Texas Children’s Hospital, Baylor College of Medicine, Department of Pediatrics, Section of Emergency Medicine, Houston, TX

## Abstract

A 5-month-old female presented to the emergency department with worsening abdominal distention and postprandial emesis. Point-of-care ultrasound (POCUS) demonstrated extensive abdominal free fluid with a large, heterogeneous mass in the suprapubic region. This was confirmed on computed tomography (CT) of the abdomen and pelvis. The patient was urgently taken to the operating room for exploratory laparotomy where a ruptured tumor was discovered. The patient was eventually diagnosed with ovarian juvenile granulosa cell tumor. POCUS can be vital in guiding the recognition of rare pediatric conditions in the emergency department and should be considered in patients where there is concern for an acute abdomen.

**Topics:**

Abdominal pain, ascites, ovarian juvenile granulosa cell tumor, point-of-care ultrasound.

[Fig f1-jetem-7-1-v8] [Fig f2-jetem-7-1-v8] [Fig f3-jetem-7-1-v8] [Fig f4-jetem-7-1-v8]

**Figure f1-jetem-7-1-v8:**
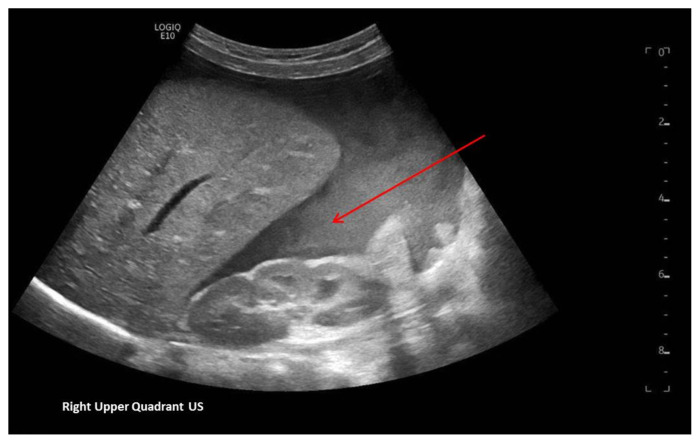


**Figure f2-jetem-7-1-v8:**
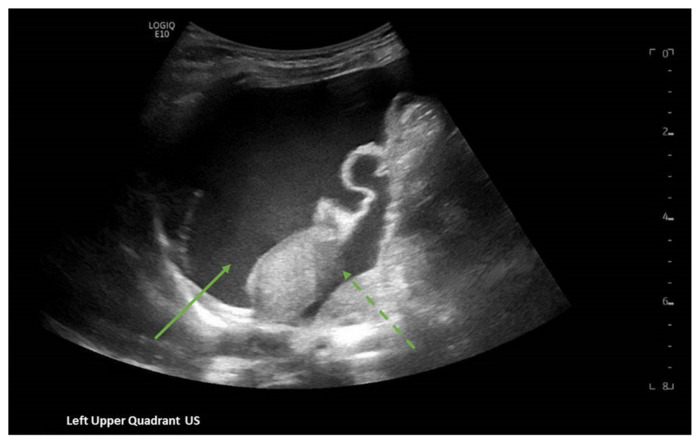


**Figure f3-jetem-7-1-v8:**
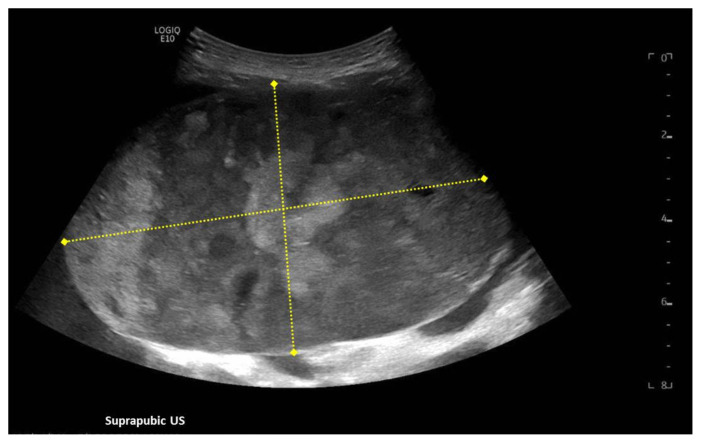


**Figure f4-jetem-7-1-v8:**
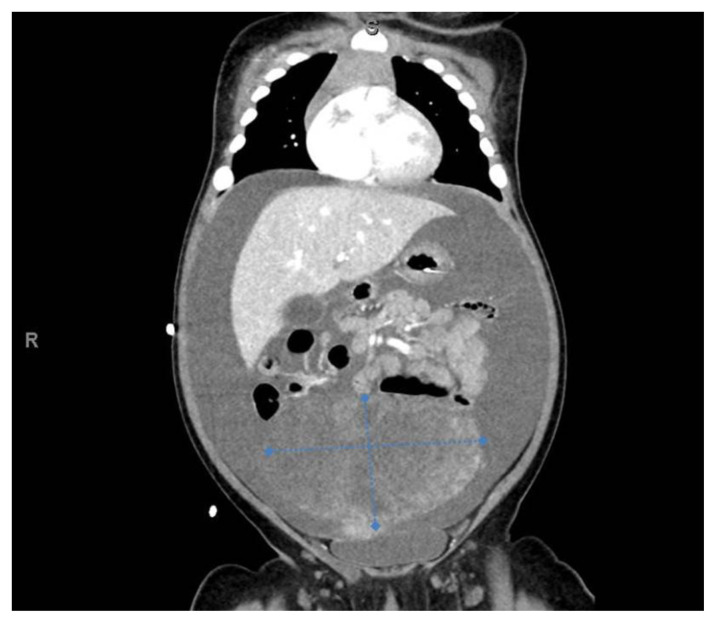


## Brief introduction

Though extremely rare, an acute abdomen due to massive ascites can be the first sign of ovarian juvenile granulosa cell tumor (JGCT) in pediatric patients.^1^ Given that many causes of acute abdomen require urgent surgical intervention, point-of-care ultrasound may expedite the diagnosis and definitive management. Prompt recognition of massive ascites due to JGCT is essential given the established knowledge that tumor rupture necessitates the involvement of a surgical subspecialist early in the patient’s clinical course.^2^

## Presenting concerns and clinical findings

A 5-month-old female presented to the emergency department with a four-day history of progressively worsening abdominal distention. The distention was associated with a one-day history of post-prandial, projectile, non-bloody, non-bilious emesis and decreased appetite. Family denied fever, diarrhea, rashes, decreased stools, or sick contacts. Clinical evaluation from outside the facility was remarkable for anemia, leukocytosis, hyponatremia, and mildly distended loops of bowel on an abdominal radiograph. Upon arrival, the patient appeared to be in moderate distress. She was grunting, pale, and noted to have a firm and distended abdomen, diffuse abdominal tenderness, and hypoactive bowel sounds. The remainder of her physical examination was unremarkable.

## Significant findings

A focused assessment with sonography in trauma (FAST) exam was performed initially to evaluate for intra-abdominal injury given the clinical picture. A phased-array ultrasound transducer was placed in sagittal orientation along the patient’s right and left flank, demonstrating extensive heterogenous fluid collections in Morrison’s pouch (red arrow), subphrenic space (solid green arrow), and splenorenal recess (dashed green arrow). To further evaluate, a phased-array transducer was placed over her pelvic area in transverse orientation, demonstrating, a large, heterogeneous mass (outlined in yellow arrows). The surgical team was promptly consulted and blood products were ordered. Although there was concern for impending hemorrhagic shock due to patient’s presenting tachycardia, the patient was hemodynamically stable enough for a CT scan of her chest, abdomen, and pelvis. The CT scan showed large-volume ascites, which exerted mass effect on all abdominal organs with centralization of bowel loops. Additionally, there was a large, 6.4 × 6.8 × 10.9-centimeter, midline pelvic mass (outlined in blue arrows).

## Patient course

The patient was urgently taken to the operating room for an exploratory laparotomy. Intraoperatively, 500 milliliters of serosanguinous fluid were drained, followed by a left salpingo-oophorectomy with removal of the actively bleeding tumor from the left fallopian tube. She was transfused with packed red blood cells during the procedure. Following the procedure, the remainder of her hospital course was uncomplicated. Pathology revealed a juvenile granulosa cell tumor.

## Discussion

There is a wide-spectrum of medical and surgical conditions that can present with acute abdominal symptomatology.^3^ Moreover, though the presence of small amounts of peritoneal fluid may be physiologic, the detection of increased free peritoneal fluid is a highly important finding in children with various potential causes, including abdominal trauma of solid or hollow viscous organs, neoplasms, sepsis, or acute inflammatory process. These patients may need aggressive resuscitation and emergent surgical intervention, necessitating early diagnosis. There is a clear utility of using ultrasonographic examination to provide important information about abdominal organs. It has also been demonstrated that the evaluation of children with an acute abdomen through point-of-care ultrasonography changes their management in a significant number of patients.^4^ Furthermore, many unique diagnoses can be revealed by using ultrasonography early in a patient’s clinical course. Although point of care ultrasound is not well studied in pediatrics, our case report highlights the importance of utilizing early POCUS in children with acute abdominal pain, while also demonstrating the potential for immediate change in management upon ultrasonographic evaluation. In this case, not only were there positive findings in the FAST exam, but ultrasonographic evaluation identified a unique etiology of the patient’s presentation, expediting her transfer to the operating room.

Although there was a lack of history concerning trauma in this patient, there was an initial clinical concern to evaluate for intra-abdominal injury with a point-of-care FAST exam due to the presenting abdominal distention and anemia. In general, the FAST, which looks at the left upper quadrant, the right upper quadrant, the pericardium, and pelvis, was first introduced as a screening tool in adult populations to assist in identifying the presence of free fluid in the abdominal cavity and pericardially.^5^,^6^ The overall objective of the FAST is to discover free intra-abdominal fluid in dependent areas. Though controversy exists regarding the use of FAST within pediatric trauma patients due to dissimilar mechanisms of injury and management from adult trauma patients, the FAST exam can be an effective screening tool for prioritizing and expediting life-saving procedures, vital diagnostic studies, subspecialty involvement, and disposition. Additionally, though mostly studied and accepted in adult medicine, a rapid ultrasound in shock and hypotension (RUSH) exam should also be considered when evaluating patients in undifferentiated shock. The RUSH exam, which should be thought of as an extension of the physical examination, incorporates the components of a FAST exam, in addition to investigating other causes, in order to identify the etiology of shock and guide management. 7,8 Nonetheless, there remains a vital role for bedside ultrasonography in pediatric populations, specifically in unstable children where the presence of abdominal fluid is likely an indication for emergency laparotomy; and though a negative bedside ultrasonographic finding cannot exclusively decrease the presence of a serious intra-abdominal process, a positive finding is enough evidence to warrant immediate exploration of the abdomen, either with CT or with surgical exploration.^5^,^6^

Though it is typically associated with isosexual development, JGCT presenting as an acute abdomen due to a ruptured tumor is the presenting symptom in about 6–10% of cases.^2^,^9^ Granulosa cell tumors (GCTs) constitute only 1% to 2% of all ovarian malignancies.^3^ These tumors are typically subdivided into the adult type and the juvenile type, with juvenile granulosa cell tumors (JGCT) representing 5% of all GCTs. Due to the hormone-active nature of this ovarian tumor, patients most commonly present with symptoms of hyperestrogenism or hyperandrogenism, such as vaginal bleeding or secretions, breast enlargement, axillary and pubic hair development, or hirsutism. Additionally, clinical symptoms, such as abdominal distention and pain, are common. JGCTs are characterized by a low malignant potential and generally have a high cure rate.

Diagnostically, there is no known specific tumor marker of JGCT.^10^ Despite the lack of diagnostic markers, the measurement of inhibin, a peptide hormone that is produced by ovarian granulosa cells, has been shown to be elevated in both adult type and juvenile type GCTs. The mainstay of treatment is surgery, which often includes a unilateral salpingo-oophorectomy in young, premenopausal women who desire to preserve fertility.^11^ In most cases of JGCTs, there is a 90–95% five-year survival with early disease. Adjunctive chemotherapy has not been found to be useful in early stages. Late recurrences may occur after the initial treatment, necessitating long-term follow up.

## Supplementary Information








