# Supplementary figures and images for: Ovarian Juvenile Granulosa Cell Tumor Case Report
Source: J Educ Teach Emerg Med. 2022 Jan 15;7(1):V8–V12. doi: 10.21980/J8035H (PMC10358864; doi:10.21980/J8035H)

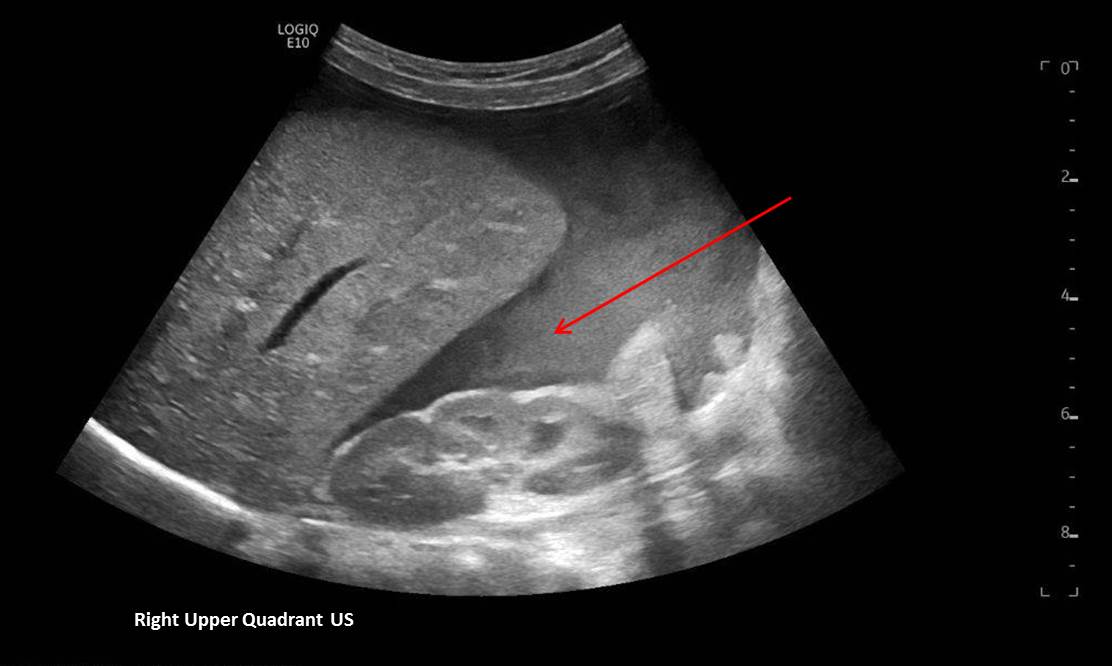

Supplement: Supplementary file 1 [file JETem-7-1-V8-supp1.jpg]

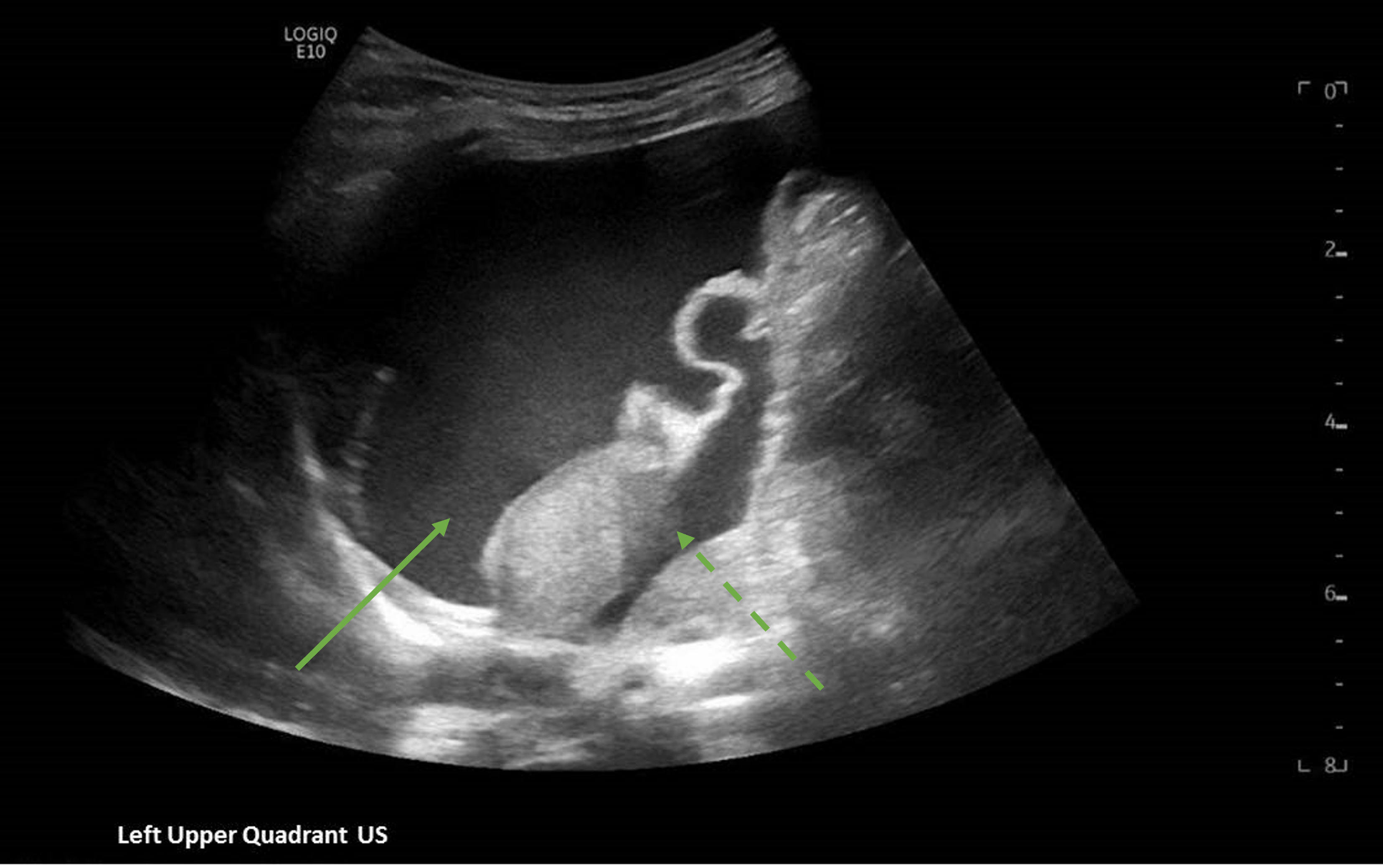

Supplement: Supplementary file 2 [file JETem-7-1-V8-supp2.jpg]

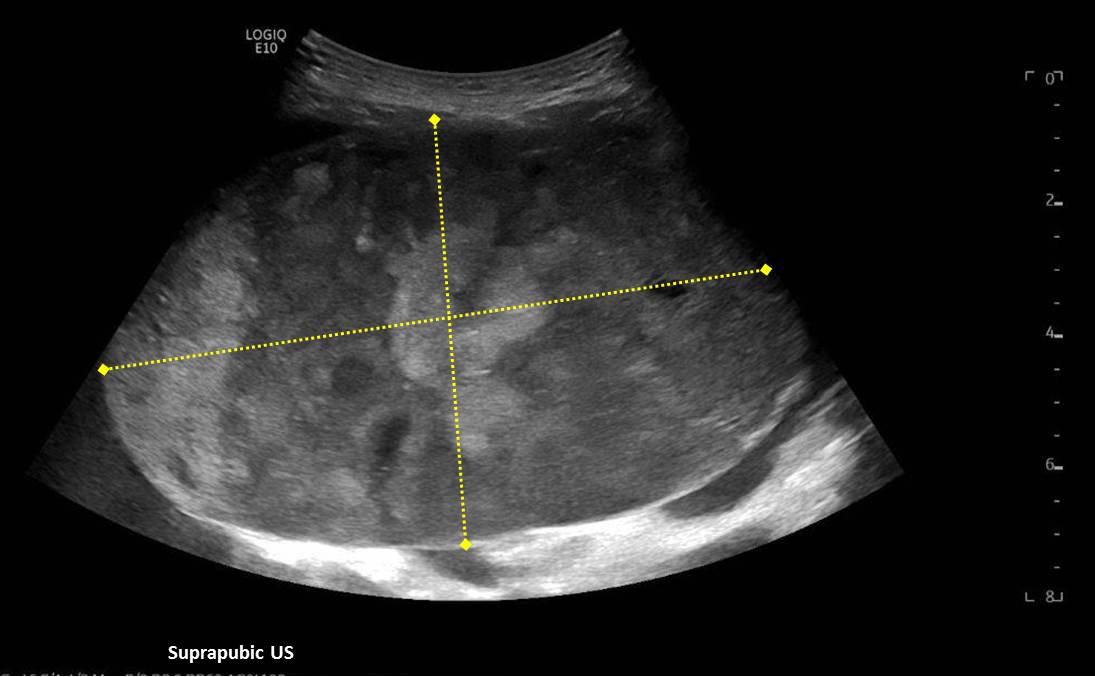

Supplement: Supplementary file 3 [file JETem-7-1-V8-supp3.jpg]

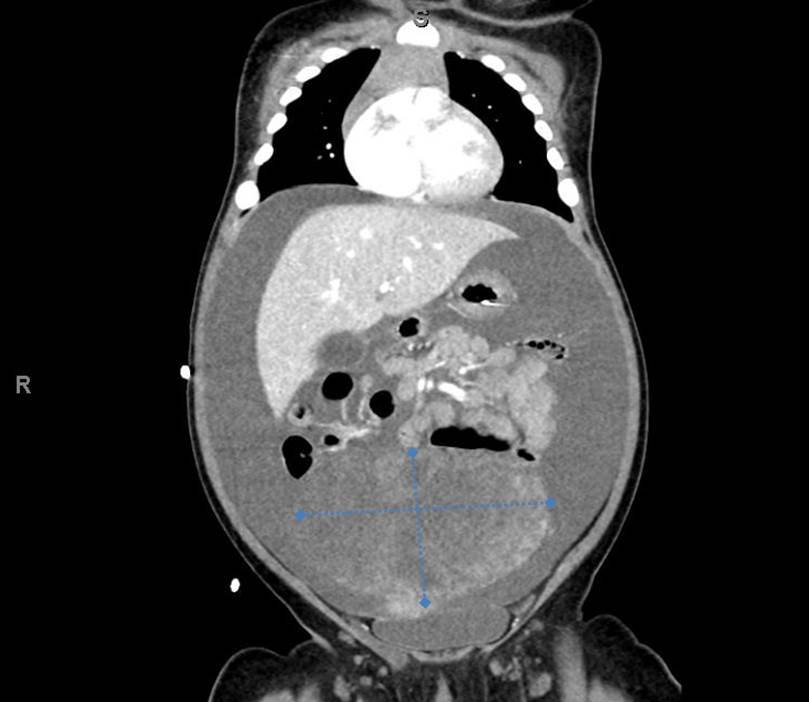

Supplement: Supplementary file 4 [file JETem-7-1-V8-supp4.jpg]
